# Correlations between Iron Load and CD4 in Adult Transfusion-Dependent Beta Thalassemia

**DOI:** 10.1155/2021/5549503

**Published:** 2021-06-17

**Authors:** Tubagus Djumhana Atmakusuma, Ralph Girson, Sukamto Koesnoe

**Affiliations:** ^1^Division of Hematology-Medical Oncology, Department of Internal Medicine Dr. Cipto Mangunkusumo General Hospital, Faculty of Medicine Universitas Indonesia, Kota Depok, Indonesia; ^2^Division of Allergy-Immunology, Department of Internal Medicine. Dr. Cipto Mangunkusumo General Hospital, Faculty of Medicine Universitas Indonesia, Kota Depok, Indonesia

## Abstract

**Background:**

Thalassemia is a hereditary disease, and severe anemia is the main phenotype of major thalassemia. Furthermore, the most important method in the management of this disease is red blood cell transfusion. Regular transfusions administered 1 or 2 times every month improve prognosis and survival. However, there is higher risk of infections and iron overload, especially in transfusion-dependent thalassemia (TDT). Infections are the second leading cause of death in adult TDT, after heart failure. Higher risk of infection is also influenced by multiple blood transfusions which causes alteration in immune response due to alloimmunization, transfusion-related infections, and iron overload. Meanwhile, iron overload in TDT alters both innate and specific immune responses. Furthermore, previous studies have shown the correlation between ferritin with CD4, but this has not been carried out in Indonesia. Therefore, this study aims to determine the correlations between iron overload (serum ferritin and transferrin saturation) and specific immune cells (CD4).

**Methods:**

This is a cross-sectional study, and a total number of 64 subjects were examined consecutively. Chest X-ray and blood sera were obtained. The total number of subjects was 64. The seromarkers HBsAg, anti-HCV, and anti-HIV were tested using the ELISA method. Serum ferritin and transferrin saturation was tested using ECLIA, and lymphocyte subsets were analyzed using flowcytometry. Meanwhile, the correlation between variables was determined using Spearman's test.

**Results:**

The results showed that 4.9% subjects were HBsAg positive, 10.7% were anti-HCV positive, and none were anti-HIV positive. There were 4 subjects with lung tuberculosis based on the 41 chest X-ray. Meanwhile, there was a weak negative and insignificant correlation between serum ferritin with CD4 (*p*=0.75; *r* = −0.04) and a weak positive and insignificant correlation between transferrin saturation with CD4 (*p*=0.133; *r* *=* 0.19).

**Conclusion:**

There were no correlations between iron overload (ferritin) and cellular immunity (CD4) in adult transfusion-dependent thalassemia.

## 1. Background

Thalassemia is a hereditary disorder on formation of alpha and beta globin chains in erythrocytes. This leads to structural abnormalities of the erythrocytes which causes anemia. Furthermore, anemia in thalassemia patients ranges from mild or moderate (thalassemia minor and intermedia) to severe (thalassemia major). Estimation has shown that there are 7% of thalassemia patients in the world population while the disease spreads mainly in the tropic and subtropic countries.[1]. In Indonesia, especially in the Cipto Mangunkusumo National Referral Hospital (RSCM) in Jakarta, there are approximately 1570 thalassemia patients undergoing regular blood transfusions.[2].

Patients with thalassemia major who suffer from anemia would require regular red blood cell transfusions to maintain growth and development. Moreover, thalassemia is a hereditary disorder; hence, patients need regular transfusions throughout lifetime. Adult thalassemia patients are classified as transfusion-dependent thalassemia (TDT), which requires regular transfusions of red blood cells once or twice every month and non-transfusion-dependent thalassemia (NTDT).[1].

Hypertransfusion extends the life expectancy and improve the life quality of patients, especially in TDT. However, it increases the risk of infection (transfusion-related infection) and also leads to iron overload. Meanwhile, both infection and iron overload are considered as risk factors for severe infections in patients with thalassemia.

Infection is the second leading cause of death after heart failure in TDT patients in Cipto Mangunkusumo Hospital (RSCM) [2]. Assadov, et al. [3] stated that patients with thalassemia possess a decrease in the specific or nonspecific immune response. Furthermore, a decrease in the number of cluster of differentiation 4 (CD4) T lymphocytes would be observed at the cellular immunity level. This is supposedly caused by multiple transfusions due to a charge of excess iron. A decreased immune response in patients with thalassemia is one of the risk factors for infection in thalassemia. In a previous study on beta thalassemia major, Kadam et al. [4] stated that the CD4 subset was lower compared to normal population. Moreover, the primary haemochromatosis study in Porto [5] showed a positive correlation between iron load and CD4/CD8 ratio, and a negative correlation between iron load and CD8. However, the mechanism of decreasing immune response in thalassemia is still ambiguous. Excessive iron load is one of the main factors which play a role through the oxidative stress pathway [5].

## 2. Methods

This is a cross-sectional study conducted in the Kiara Outpatient Thalassemia Unit, RSCM, in October 2016. Samples were collected consecutively from adult patients with thalassemia who went to the clinic and received blood transfusions every month. The exclusion criteria were patient refusal or if the patient had undergone splenectomy. This study was approved by the Ethics Committee of the Faculty of Medicine, University of Indonesia, with clearance number 837/UN2.F1/ETIK/2016. Appropriate consent was obtained from all subjects.

The medical history and physical and laboratory examination of peripheral blood from the subject venous blood were taken at the Laboratory of Clinical Pathology, RSCM. This includes a complete peripheral blood test, hepatitis B surface antigen (HBsAg), anti-hepatitis C virus (anti-HCV), anti-human immunodeficiency virus (anti-HIV), serum ferritin, transferrin saturation, high-sensitivity C Reactive Protein (hsCRP), and CD4 and CD8 cells. A complete blood count was performed using Sysmex® XN-1000 from Sysmex™ Corporation, and assay for CRP was carried out using Roche™ Cobas® c311. Furthermore, Seromarkers HBsAg, anti-HCV, and anti-HIV tests were performed by the ELISA method using Abbott™ Architect® i1000/i2000 devices. The serum ferritin and transferrin saturation tests were also performed by the ECLIA method using Architect® C4000/8000 by Abbott™, and flowcytometry was conducted using FACS Calibur® by Becton Dickinson™ company.

The chest x-ray examination was performed in the RSCM radiology department. Furthermore, data were recorded on the study worksheet and processed into a scatter diagram while the correlation was tested using Spearman correlation.

## 3. Results

Out of the 78 transfusion-dependent beta thalassemia adult patients recruited, 14 were excluded because 12 patients had undergone splenectomy and 2 refused to participate. Therefore, 64 subjects agreed to participate in the blood test. However, only 41 subjects underwent chest x-ray examinations.

There were 33 female (52%) and 31 male subjects (48%) with ages ranging from 18 to 37 years, with a median of 22 years of age (Table 1). In this study, there were 27 subjects (42.2%) with *β*-thalassemia major and 37 subjects (57.8%) with hemoglobin *E* (HbE) thalassemia.

Chronic infectious diseases documented include tuberculosis (TBC), HIV, and chronic hepatitis. At chest X-ray examination, there were 4 subjects (9.7%) out of 41 screened subjects with pulmonary tuberculosis while one subject received anti-tuberculosis drug therapy. The patients with hepatitis disease consisted of 8 people (12.5%) out of 64 patients. Moreover, 1 patient had hepatitis B (1.6%) positive, 5 had hepatitis C (7.8%), and 2 were with hepatitis B and C (3.1%). There were no results of patients with a history of human immunodeficiency virus (HIV) disease. The data for serum ferritin and transferrin saturation are shown in Table 2.

### 3.1. Correlation between Serum Ferritin with CD4 Count in Adult Transfusion-Dependent Beta Thalassemia Patients

**Figure 1:**
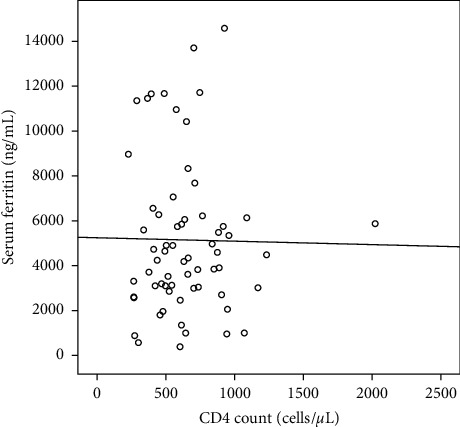
Scatter diagram correlation between serum ferritin with CD4.

Spearman's correlation analysis was conducted using the results of the *r* value = −0.04 with a significance level of 0.75 (Figure 1, Table 3)

In Table 2, a correlation (*r*) value of −0.04, with a significance value of 0.75 (*p* > 0.05), was obtained. Therefore, there is no correlation between serum ferritin with CD4.

### 3.2. Correlation between Transferrin Saturation with CD4 Count in Adult TDT Patients

**Figure 2:**
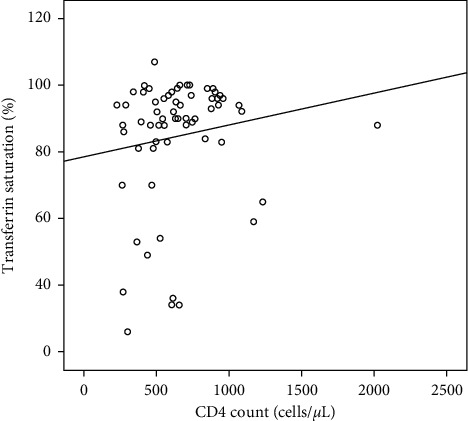
Scatter diagram of the correlation between transferrin saturation with CD4.

Analysis of Spearman's correlation analysis was performed using the correlation *r* value of 0.19 with a significance level of 0.133 (Figure 2, Table 4).

Based on Table 3, *r* correlation values of 0.19 showed a weak positive correlation. The *p* value of 0.133 (*p* > 0.05) showed that there was no significant correlation between transferrin saturation and CD4.

## 4. Discussion

The results showed a weak positive, and there was no significant correlation between serum ferritin with CD4. Similarly, iron load markers showed transferrin saturation with CD4, and the results showed an insignificant and a weak negative correlation. The correlation between excess iron load with CD4/CD8 ratio as established by Porto et al. [5] who stated that, in a hereditary haemochromatosis population, excess iron load is not due to transfusion. However, the difference in results is due to variation in the population which is the charge of excess iron due to hypertransfusion in subjects. Meanwhile, more factors contributed to iron overload and immune deficiency in our patients compared to haemochromatosis patients in Porto et al.'s [5] study.

A previous study with similar aims by Amrita et al. [6] recruited 36 TDT patients and showed correlation between ferritin serum and CD4 counts [6], but this study had different results. This is due to larger sample size (64 vs. 36) and greater genetic diversity in subjects. In addition, the study by Amrita et al. was conducted at a regional referral center, while this study was conducted in a national referral center. Therefore, this study had a greater genetic diversity due to subjects referred from nationwide thalassemia centers.

The results of this study are in accordance with a previous study by Arseno et al. [7] in pediatric TDT patients which had no correlation between ferritin levels and CD4 cell count [7]. Meanwhile, Arseno et al. stated that the absolute number of CD4 count has no significant role in TDT. However, immune dysfunction in children with TDT is caused by defect in the T-lymphocyte function rather than by the decrease of absolute T-cell count [7].

Meanwhile, the correlation between iron load and cellular immunity was studied by Hagag et al. [8] which describes a significant correlation between serum ferritin with CD4 and CD8. The study population consisted of nonsplenectomized children with serum ferritin >1000 ng/mL and was compared to a normal population. However, other studies show differences in the humoral immune response where quantitative measurements are not included. Serum ferritin and transferrin saturation were used as the iron load marker in this study. Ferritin is an intracellular-extracellular iron-binding protein which refers to the iron-containing molecules while the term apoferritin is used for the iron-free molecules [9]. Transferrin saturation is obtained from the calculation of serum iron to total iron-binding capacity ratio, and the results showed that both had insignificant data. Although there were some differences, it is not yet concluded that the saturation transferrin provides better monitoring on hematopoiesis. Furthermore, the charge of excess iron is best monitored by measuring redox-active iron such as non-transferrin-bound iron (NTBI) and labile plasma iron (LPI) in plasma and labile iron pools (LIP) in the cellular cytoplasm.

Ferritin is an acute phase reactant; therefore, acute or chronic infection interfered with the results. However, in this study, the subjects had normal median hsCRP levels. Therefore, infection can be excluded, and the increase in serum ferritin is considered to be solely caused by iron overload.

The factors that caused insignificant correlation in our study were high levels of serum ferritin, iron chelation, and vitamin *E* treatments. Although average ferritin was high in subjects, the effects of iron chelation occurred in a short period of time. Meanwhile, previous studies stated that administration of iron chelation poses an effect on CD8 and directly reduces inflammation [10, 11]. Oxidants decrease immune response; therefore, administration of vitamin *E* has a role in improving cellular immune response [12]. The subject examined had both vitamin *E* and iron chelation as routine drug treatment for thalassemia.

One of the factors to be considered is the large percentage of patients with splenomegaly (89.1%). The spleen plays an important role in the host defense against invading pathogens [13]. A previous study by Chu et al. [14] in cirrhotic patients with splenomegaly showed that there is an impairment of cellular immune function and T-lymphocytes subset while the number of CD3+, CD4+, and CD8+ increases significantly [14]. In addition, a previous study in thalassemia patients showed that splenectomized patients had higher absolute lymphocyte subset count [15].

The relationship between iron chelation agents and CD4 count involves a complex mechanism. Iron is important for the survival of organisms including pathogens [16]. However, iron overload hinders optimal immune response. Adequate iron chelation is essential to defend the host against a pathogenic microorganism [17]. All chelating drugs when used appropriately are sufficient to prevent bacterial growth. Meanwhile, Kontoghiorghes [17] stated that deferiprone has the highest long-term antimicrobial effect. In addition, a previous study by Del Vecchio et al. [18] showed that deferiprone normalizes the CD4/CD8 ratio after treatment. Hence, this study showed that the iron chelating regiment varied and large percentage of patients were unable to achieve optimal chelation target due to budget restriction in medications. Further studies are required to determine the role of specific iron chelating therapy in modulating the host immune response. Based on the results, complex interplaying factors changed the immunological response of patients with TDT.

The limitations of this study are the cross-sectional study design and the conditions of patients were different from one another which affected the results. Furthermore, variations in spleen size and iron chelating regiment also influenced the results.

## 5. Conclusions

There was no significant correlation between serum ferritin and transferrin saturation with CD4 in adults with transfusion-dependent beta thalassemia.

## Figures and Tables

**Table 1:** Baseline characteristics of study subjects.

Variables	Gender		*N* = 64
	Male (*n* = 31 [48.4%])	Female (*n* = 33 [51.6%])	
Age (years)			
Median (range)	22 (18, 37)	21 (19, 33)	22 (18, 37)

Type of thalassemia			
*Thalassemia β* major	14 (45.2)	13 (39.4)	27 (42.2)
*Thalassemia β* HbE	17 (54.8)	20 (60.6)	37 (57.8)

Chronic infectious diseases			
HIV	0	0	0
Hepatitis B	0	1 (3.0)	1 (1.6)
Hepatitis C	5 (16.1)	0	5 (7.8)
Hepatitis B and C	0	2 (6.1)	2 (3.1)
TBC (*N* = 41)	2 (6.5)	2 (6.1)	4 (9.8)

First transfusion			
Less than 6 years	24 (77.4)	26 (78.8)	50 (78.1)
More than 6 years	7 (22.6)	7 (21.2)	14 (21.9)

Number of transfusions			
More than 1 time per month	21 (67.7)	24 (72.7)	45 (70.3)
One month	10 (32.3)	9 (27.3)	19 (29.7)

Iron-chelating drug			
Mono	26 (83.9)	30 (90.9)	56 (87.5)
Combination	5 (16.1)	3 (9.1)	8 (12.5)

Regularity of taking medicine			
Not a routine	24 (77.4)	26 (78.8)	50 (78.1)
Routine	7 (22.6)	7 (21.2)	14 (21.9)

Enlargement of the spleen (splenomegaly)			
Normal	4 (12.9)	3 (9.1)	7 (10.9)
Splenomegaly	27 (87.1)	30 (90.9)	57 (89.1)

Facies Cooley			
No	16 (51.6)	18 (54.5)	34 (53.1)
Yes	15 (48.4)	15 (45.5)	30 (46.9)

Hemoglobin (g/dL)^∗^	8.2 (1.3)	7.8 (1.1)	8.01 (1.2)
The mean (SB)			

Leukocyte (10^3/uL)	4.9 (2.3, 9.5)	4.7 (1.7, 11.6)	4.9 (1.7, 11.6)
Median (range)			

Neutrophils	2820 (920, 5460)	2770 (730, 7160)	2795 (730, 7160)
Median (range)			

Platelets (10^3/uL)	135 (41, 331)	130 (52, 366)	131 (41, 366)
Median (range)			

Hs CRP (mg/L)	2.0 (0.4, 9.5)	1.6 (0.6, 8.9)	1.8 (0.4, 9.5)
Median (range)			

**Table 2:** Iron status.

Variables	Median (interquartile range)
Ferritin (ng/mL)	4,595.00 (3,233.25)
Transferrin saturation (%)	91 (16)

**Table 3:** Correlation between serum ferritin with CD4 count in adult TDT patients.

Variables	*r*	*p (Spearman's)*
*Serum ferritin* with CD4	−0.04	0.75

**Table 4:** Correlation between transferrin saturation with a CD4 cell count in adult patients with transfusion-dependent beta thalassemia.

Variables	*r*	*p (Spearman's)*
Transferrin saturation with CD4 count	0.19	0.133

## Data Availability

Additional data can be requested by contacting the corresponding author through the e-mail address provided.
